# Phonon Boost Effect on the *S*^±^-wave Superconductor with Incipient Band

**DOI:** 10.1038/s41598-019-40536-3

**Published:** 2019-03-07

**Authors:** Yunkyu Bang

**Affiliations:** 1 Asia Pacific Center for Theoretical Physics, Pohang, Gyeongbuk 790-784 Korea; 2 Department of Physics, POSTECH, Pohang, Gyeongbuk 790-784 Korea

## Abstract

We showed that the all phonons – not only forward-scattering phonon but also local (all-momentum-scattering) phonon – contribute to boosting *Tc* of the *S*^±^-wave pairing state in the incipient band model. In particular, when the incipient band sinks deeper, the phonon boost effect of the local phonon increases and becomes as effective as the one of the forward-scattering phonon. Our finding implies that all interface phonons from the SrTiO_3_ substrate – not only the 90 *meV* Fuchs-Kliewer (F-K) phonon but also the 60 *meV* F-K phonon – as well as all intrinsic phonons of the FeSe monolayer itself, regardless of their degree of “*forwardness*”, should contribute to increase *Tc* of the FeSe/STO monolayer system. This all-phonon boost mechanism should universally work in all heavily doped (either by holes or by electrons) Iron-based superconductors.

## Introduction

The discovery of the FeSe/SrTiO_3_ monolayer system (*T*_*c*_ ≈ 60–100 *K*)^1–3^ is posing a serious challenge to our understanding of the Iron-based superconductors (IBS)^4–6^. As to the origin of such a high *T*_*c*_ value, it is widely accepted that the forward-scattering phonon, penetrating from the SrTiO_3_ substrate, is the key booster for increasing *T*_*c*_^7–11^. Indeed, Lee *et al*.^7^ have measured the replica band shifted by about 90 *meV* downward from the original electron band, and it was claimed to be a smoking-gun evidence for the presence of the forward-scattering phonon coupled to the conduction band electrons in the FeSe monolayer. Subsequently, the high-resolution electron energy loss spectroscopy (HREELS) measurement^12^ has directly measured the 90 *meV* and 60 *meV* Fuchs-Kliewer (F-K) interface phonon modes in the FeSe/SrTiO_3_(STO) system^12^ besides several lower energy bulk phonon modes of FeSe layer. However, this HREELS experiment itself has no information about whether the observed F-K modes (both 90 *meV* and 60 *meV* modes) are forward-scattering or all-momentum scattering phonons.

Then, recently, Sawatzky and coworkers^13^ have pointed that the replica band observed in the Angle Resolved Photo-Emission Spectroscopy (ARPES) experiment^7^ is not the evidence of a forward-scattering phonon coupled to the conduction electrons of the FeSe layer but a consequence of the kinematics of the escaping electrons in the ARPES measurement. The main point of this criticism is that it is true that there exists a F-K phonon mode of the energy ~90 *meV*^12^, but there is no evidence that this phonon is a small-momentum exchange (forward-scattering) phonon which produces the replica band by coupling to the main conduction band electrons of the FeSe layer; it may couple but via all-momentum exchange and in this case no replica band can be formed.

In this paper, we studied the effect of the generic types of phonons on the superconducting (SC) instability of the *s*^±^-gap symmetry in the incipient band model. The incipient band model^14,15^ has been introduced to study the superconductivity of the so-called heavily electron-doped iron selenide (HEDIS) compounds. All HEDIS compounds have the missing hole pocket (Fermi surface) because the hole band sinks below Fermi level by about ~60–90 *meV* due to the heavy electron doping, hence also called as an incipient band. Despite missing hole pocket, these compounds have achieved surprisingly high *T*_*c*_ values: A_*x*_Fe_2−*y*_Se_2_ (A = K, Rb, Cs, Tl, etc.) (*T*_*c*_ ≈ 30–40 *K*)^16–18^ and (Li_1−*x*_Fe_*x*_OH)FeSe (*T*_*c*_ ≈ 40 *K*)^19^, and the FeSe/SrTiO_3_ monolayer system (*T*_*c*_ ≈ 60–100 *K*)^1–3^. Therefore, the origin of these high *T*_*c*_ values of the HEDIS compounds or the incipient band system is a challenging mystery to be understood, and in particular it is curious to know whether the incipient band plays any active role to enhance *T*_*c*_.

As to these questions, our study in current paper has found that the all phonons–not only forward-scattering phonon but also local (all-momentum-scattering) phonon–contribute to boost *T*_*c*_ of the incipient band superconductor. In particular, we showed that as the incipient band sinks deeper, the phonon boost effect of both types of phonon becomes indistinguishably similar from one another. It means that the sunken hole band plays an active role to turn otherwise useless phonons (all-momentum scattering phonons) into useful pairing glues. Our finding implies that all phonons^12^, both interface phonons from the STO substrate (the 90 *meV* and 60 *meV* F-K phonons) and intrinsic phonons of the FeSe monolayer itself should contribute to increase *T*_*c*_ of the FeSe/STO monolayer system. This all-phonon boost mechanism should work in all heavily doped (either by holes or by electrons) Iron-based superconductors.

## Results

### Phonon Boost Mechanism

The underlying reason for this surprising result is because the relative size of Δ^+^-gap and Δ^−^-gap of the *s*^±^-wave pairing state in the incipient band superconductor is generically not equal, and this disparity of gap size grows as the incipient band sinks deeper. To illustrate the consequence of this effect, let us recollect the general principle of a phonon contribution to the unconventional superconductor with a sign-changing order parameter (OP) Δ(*k*), in general. In the BCS pairing theory with pairing interactions of *V*_*sf*_(*k*, *k*′) and *V*_*ph*_(*k*, *k*′), the gap equation at *T*_*c*_ with a gap function Δ(*k*) has the following structure

1 $$\begin{array}{rcl}{\rm{\Delta }}(k) & = & -\,{\sum }_{k^{\prime} }{V}_{sf}(k,k^{\prime} ){\rm{\Delta }}(k^{\prime} ){\chi }_{sf}(T)\\  &  & -\,{\sum }_{k^{\prime} }{V}_{ph}(k,k^{\prime} ){\rm{\Delta }}(k^{\prime} ){\chi }_{ph}(T),\end{array}$$

where *V*_*sf*_(*k*, *k*′)(>0) is a repulsive spin-fluctuation mediated interaction and *V*_*ph*_(*k*, *k*′)(<0) is an attractive phonon interaction. *χ*_*sf*(*ph*)_(*T*) are the pair susceptibilities defined as ${\chi }_{sf}(T)=N\mathrm{(0)}{\int }_{-\,{{\rm{\Lambda }}}_{sf}}^{{{\rm{\Lambda }}}_{sf}}d\xi \frac{\tanh (\frac{\xi }{2T})}{2\xi }$ and ${\chi }_{ph}(T)=N\mathrm{(0)}{\int }_{-\,{\omega }_{D}}^{{\omega }_{D}}d\xi \frac{\tanh (\frac{\xi }{2T})}{2\xi }=N\mathrm{(0)}\varphi (T,{\omega }_{D})$, respectively, where *ϕ*(*T*, *ω*_*D*_) is the result of the integral ${\int }_{-\,{\omega }_{D}}^{{\omega }_{D}}d\xi \ldots$. Assuming that the gap symmetry is already determined by the primary pairing interaction *V*_*sf*_(*k*, *k*′), the second term of Eq. (1) defines the additional contribution from the phonon interaction *V*_*ph*_(*k*, *k*′) to the total pairing as

2 $${\sum }_{k^{\prime} }{V}_{ph}(k,k^{\prime} ){\rm{\Delta }}(k^{\prime} ){\chi }_{ph}(T)=\varphi (T,{\omega }_{D})N\mathrm{(0)\;  < }\,{V}_{ph}(k,k^{\prime} ){\rm{\Delta }}(k^{\prime} {) > }_{k^{\prime} },$$

where <···> _*k*_ means the Fermi surface (FS) average. For example, the contribution of the local Einstein phonon interaction *V*_*ph*_(*k*, *k*′) = *V*_0_ to the *d*-wave gap, ${{\rm{\Delta }}}_{d}(k) \sim (\cos \,{k}_{x}-\,\cos \,{k}_{y})$, would be null because

3 $${V}_{0} < {{\rm{\Delta }}}_{d}(k^{\prime} {) > }_{k^{\prime} }=0.$$

On the other hand, if the phonon potential *V*_*ph*_(*k*, *k*′) represents a forward-scattering phonon, namely, a dominantly stronger potential when the angle (*θ*) between $\overrightarrow{k}$ and $\overrightarrow{k^{\prime} }$ is smaller than a certain angle, say, |*θ*| < *π*/4, it is obvious that

4 $$ < {V}_{ph}(k,k^{\prime} ){{\rm{\Delta }}}_{d}(k^{\prime} ){ > }_{k^{\prime} }\ne 0,$$

so that the attractive phonon interaction cooperates with the repulsive spin fluctuation interaction *V*_*sf*_(*k*, *k*′) in Eq. (1) to boost *T*_*c*_ of the *d*-wave pairing Δ_*d*_(*k*)^20^.

The same mechanism would work for the *s*^±^-wave pairing state. Here the relevant quantity is

5 $${\sum }_{k^{\prime} }{V}_{ph}(k,k^{\prime} )[{{\rm{\Delta }}}^{+}(k^{\prime} )+{{\rm{\Delta }}}^{-}(k^{\prime} )],$$

where Δ^+^(*k*) and Δ^−^(*k*) are the gap functions of the hole and electron bands, respectively, and have opposite signs each other. Let us first consider the special case of an equal size of OPs |Δ^+^(*k*′)| = |Δ^−^(*k*′)|. Then the contribution of the local Einstein phonon interaction *V*_*ph*_(*k*, *k*′) = *V*_0_ to the *s*^±^-wave pairing is proportional to

6 $${V}_{0} < [{{\rm{\Delta }}}^{+}(k^{\prime} )+{{\rm{\Delta }}}^{-}(k^{\prime} )]{ > }_{k^{\prime} }=0,$$

hence the phonon boost effect of the ordinary Einstein phonon is null for this non-generic case of the *s*^±^-wave state with the equal size of OPs |Δ^+^(*k*′)| = |Δ^−^(*k*′)|. On the other hand, with a forward-scattering phonon, namely, a dominantly stronger potential when ${\rm{\Delta }}k=|\overrightarrow{k}-\overrightarrow{k^{\prime} }|$ is smaller than the typical distance between the hole band and electron band in the Brillouin zone (BZ), say, Δ*k* < *Q* = |(*π*, *π*)|, it is obvious that

7 $$ < {V}_{ph}(k,k^{\prime} )[{{\rm{\Delta }}}^{+}({k^{\prime} }_{h})+{{\rm{\Delta }}}^{-}({k^{\prime} }_{e}{)] > }_{k^{\prime} }\ne \mathrm{0,}$$

for any fixed momentum “*k*” either on the hole FS or on the electron FS. As a result, the forward-scattering phonon interaction boosts the *T*_*c*_ of the *s*^±^-wave state with an equal size of OPs |Δ^+^(*k*′)| = |Δ^−^(*k*′)|^21^ just as in the case of the *d*-wave state.

Having illustrated the special case of an equal size of OPs in the *s*^±^-wave state, it is easy to estimate the phonon boost effect on the general *s*^±^-wave pairing state with unequal sizes of OPs |Δ^+^(*k*′)| ≠ |Δ^−^(*k*′)|. Obviously, the result of Eq. (6) with a local phonon would not be zero if |Δ^+^(*k*′)| ≠ |Δ^−^(*k*′)|. Hence, we can predict that even a local Einstein phonon can boost the *T*_*c*_ of the general *s*^±^-wave pairing state. In the case of a forward-scattering phonon, we can see that the result of Eq. (7) would be further deviated from zero, hence the phonon boost effect is even more enhanced. The most general expression of calculating the phonon boost effect for the *s*^±^-wave pairing state can be written as,

8 $$ < {V}_{ph}(k,k^{\prime} )[{{\rm{\Delta }}}^{+}({k^{\prime} }_{h}){\chi }_{ph}^{h}(T)+{{\rm{\Delta }}}^{-}({k^{\prime} }_{e}){\chi }_{ph}^{e}(T)]{ > }_{k^{\prime} }\ne 0.$$

**Figure 1:**
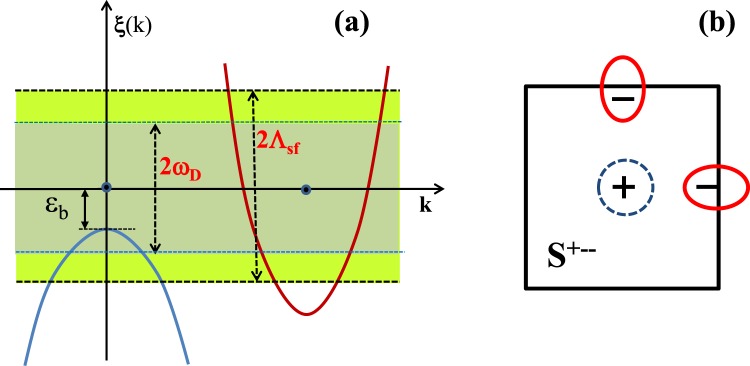
(**a**) A typical incipient band model with *ω*_*D*_ < Λ_*sf*_. Λ_*sf*_ is the spin fluctuation energy cutoff and *ω*_*D*_ is the phonon energy cutoff. *ω*_*D*_ can be larger or smaller than *ε*_*b*_. (**b**) Schematic picture of the Fermi surfaces and the incipient ${s}_{he}^{+-}$-wave solution. The hole band has no FS and the dotted circle at Γ point only indicates the SC gap character.

As mentioned already, the sizes of the gap |Δ^+^| and |Δ^−^| are not equal in general when *N*_*h*_(0) ≠ *N*_*e*_(0)^22^. In addition to that, in the case of the incipient band model^14,15,23^, the pair susceptibilities ${\chi }_{ph}^{h(e)}$ can be very different because the integration range of each band is different such as ${\chi }_{ph}^{h} \sim {\int }_{-\,{\omega }_{D}}^{-\,{\varepsilon }_{b}}d\xi \cdots$ and ${\chi }_{ph}^{e} \sim {\int }_{-\,{\omega }_{D}}^{{\omega }_{D}}d\xi \cdots$, respectively, where *ε*_*b*_ is the incipient band distance (see Fig. 1). This effect would enhance the disparity between the hole band and electron band contributions in Eq. (8) even in the case when *N*_*h*_(0) = *N*_*e*_(0). Therefore, in the incipient band superconductor, Eq. (8) can be largely deviated from zero and the total contribution to the pairing instability should be

9 $${\sum }_{k}[ < {V}_{ph}(k,k^{\prime} )[{{\rm{\Delta }}}^{+}({k^{\prime} }_{h}){\chi }_{ph}^{h}(T)+{{\rm{\Delta }}}^{-}({k^{\prime} }_{e}){\chi }_{ph}^{e}(T)]{ > }_{k^{\prime} }] < 0,$$

assuming an attractive phonon interaction, *V*_*ph*_(*k*, *k*′) < 0. As a result, we expect that any realistic phonons would contribute to increasing the *T*_*c*_ of the *s*^±^-wave state in the incipient band model, regardless of whether the phonon potential *V*_*ph*_(*k*, *k*′) is from a forward-scattering phonon or a local (all-momentum-scattering) phonon. However, Eq. (9) also tells us that a backward-scattering phonon, that is stronger for larger-momentum exchange, would suppress *T*_*c*_ instead, but we will not consider such an unrealistic phonon. In the following, we studied this phonon-boost effect quantitatively with numerical calculations of *T*_*c*_ of a minimal incipient band model, and confirmed that our speculation is indeed true.

### Incipient Band Model

The minimal incipient band model is depicted in Fig. 1. The hole band is sunken below the Fermi level by *ε*_*b*_, hence it has no FS, and the two electron bands located at X and Y points are treated as one electron band in the minimal two band model. For the pairing interactions, we assumed that the spin fluctuation mediated repulsive interaction *V*_*sf*_(*k*, *k*′)(>0) is operating within the cutoff energy scale Λ_*sf*_ and the phonon mediated attractive interaction *V*_*ph*_(*k*, *k*′)(<0) is operating within the cutoff energy scale *ω*_*D*_ (<Λ_*sf*_). This model has the incipient *s*^±^-wave solution as the most stable SC ground state^14,15^ as depicted in Fig. 1(b).

For simplicity of calculations but without loss of generality, we simplify the momentum dependent pairing interactions *V*_*sf*(*ph*)_(*k*, *k*′) as the 2 × 2 matrix potentials depicting the intra-band and inter-band interactions ${V}_{sf(ph)}^{ab},(a,b=h,e)$, then the *T*_*c*_-equation of the incipient two band model is written as

10 $$\begin{array}{rcl}{{\rm{\Delta }}}_{h} & = & [{V}_{sf}^{hh}{\chi }_{sf}^{h}+{V}_{ph}^{hh}{\chi }_{ph}^{h}]{{\rm{\Delta }}}_{h}+[{V}_{sf}^{he}{\chi }_{sf}^{e}+{V}_{ph}^{he}{\chi }_{ph}^{e}]{{\rm{\Delta }}}_{e},\\ {{\rm{\Delta }}}_{e} & = & [{V}_{sf}^{ee}{\chi }_{sf}^{e}+{V}_{ph}^{ee}{\chi }_{ph}^{e}]{{\rm{\Delta }}}_{e}+[{V}_{sf}^{eh}{\chi }_{sf}^{h}+{V}_{ph}^{eh}{\chi }_{ph}^{h}]{{\rm{\Delta }}}_{h}\end{array}$$

where the pair susceptibilities are defined as

11 $$\begin{array}{ccc}{\chi }_{sf(ph)}^{h}(T) & = & -\,\frac{{N}_{h}}{2}{\int }_{-\,{{\rm{\Lambda }}}_{sf(ph)}}^{-\,{\varepsilon }_{b}}\frac{d\xi }{\xi }\,\tanh (\frac{\xi }{2T})\\ {\chi }_{sf(ph)}^{e}(T) & = & -\,{N}_{e}{\int }_{-\,{{\rm{\Lambda }}}_{sf(ph)}}^{{{\rm{\Lambda }}}_{sf(ph)}}\frac{d\xi }{\xi }\,\tanh (\frac{\xi }{2T})\end{array}$$

where Λ_*ph*_ = *ω*_*D*_. Obviously ${\chi }_{ph}^{h}(T)=0$ when *ω*_*D*_ < *ε*_*b*_.

### Numerical Calculations

**Figure 2:**
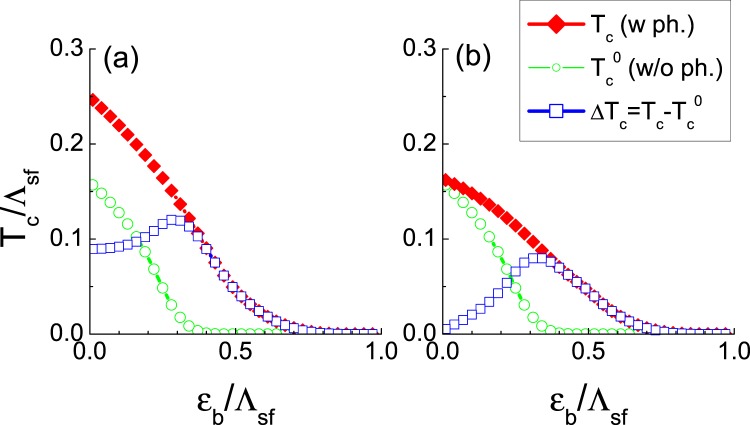
(**a**) Calculated *T*_*c*_*vs ε*_*b*_ of Eq. (9). Green symbols are ${T}_{c}^{0}$ without the phonon interaction, i.e. ${V}_{ph}^{ab}=0$, but only with the spin fluctuation mediated repulsive potential $\sqrt{{N}_{e}{N}_{h}}{V}_{sf}^{he(eh)}=1.5$ and ${N}_{e(h)}{V}_{sf}^{ee(hh)}=0.4$. Red diamond symbols are *T*_*c*_ including the additional forward-scattering phonon interaction ${N}_{e}{V}_{ph}^{ee(hh)}=-0.5$ and $\sqrt{{N}_{h}{N}_{e}}{V}_{ph}^{he(eh)}=0.0$. The blue square symbols are the net phonon boost effect ${\rm{\Delta }}{T}_{c}={T}_{c}-{T}_{c}^{0}$. (**b**) The same calculations as (**a**) but with the all-momentum-scattering local phonon interaction ${N}_{e}{V}_{ph}^{ee(hh)}=\sqrt{{N}_{h}{N}_{e}}{V}_{ph}^{he(eh)}=-\,0.5$.

First, we calculated the ${T}_{c}^{0}$ as a function of *ε*_*b*_ without phonon interaction, i.e. ${V}_{ph}^{ab}=0$, but only with spin fluctuation mediated repulsive potential ${V}_{sf}^{ab}(\, > \,0)$. We used representative values of the spin-fluctuation mediated repulsive potential $\sqrt{{N}_{e}{N}_{h}}{V}_{sf}^{he(eh)}=1.5$ and ${N}_{e(h)}{V}_{sf}^{ee(hh)}=0.4$, and assumed *N*_*e*_ = *N*_*h*_ in all our calculations in this paper. The result of ${T}_{c}^{0}$ is the green circle symbols in Fig. 2(a,b). ${T}_{c}^{0}$ gradually decreases as *ε*_*b*_ increases as expected^14,15^.

Now we turn on the attractive phonon interaction ${V}_{ph}^{ab}(\, < \,0)$ in addition to the repulsive spin fluctuation interaction ${V}_{sf}^{ab}$. We assume the Debye frequency *ω*_*D*_ = 0.5Λ_*sf*_ for all calculations in this paper. We first test a forward-scattering phonon, i.e. ${N}_{e}{V}_{ph}^{he(eh)}\,=\,0.0$ and ${N}_{e}{V}_{ph}^{ee(hh)}=-\,0.5$. The results of the calculated *T*_*c*_ are the solid diamond symbols (red) in Fig. 2(a). Apparently, *T*_*c*_ is enhanced almost uniformly from ${T}_{c}^{0}$. To see more details, we extracted the net amount of the phonon boost effect of *T*_*c*_ as ${\rm{\Delta }}{T}_{c}={T}_{c}-{T}_{c}^{0}$, which is plotted by the blue square symbols in Fig. 2(a). Interestingly, the phonon boost effect of the purely forward-scattering phonon shows an interesting dependence on *ε*_*b*_; it peaks roughly when the ${T}_{c}^{0}$ collapses to zero. This behavior tells us a complicated role of the phonon interaction for the total pairing instability. First, the fact that Δ*T*_*c*_ is always >0 definitely proves that the phonon interaction cooperates with the spin-fluctuation mediated interaction to increase *T*_*c*_. However, the fact that Δ*T*_*c*_ has a maximum peak near when ${T}_{c}^{0}$ approaches zero indicates that there is also a subtle competition (or cancellation) between the attractive phonon interaction ${V}_{ph}^{ee(hh)}(\, < \,0)$ and the repulsive spin-fluctuation mediated interaction ${V}_{sf}^{ee(hh)}(\, > \,0)$. However, this competition is confined within the intra-band interactions for the forward-scattering phonon and rather weak. Other than this detail, the results of Fig. 2(a) confirms the well known concept of the forward-scattering phonon boost effect of *T*_*c*_ in the unconventional superconductor with a sign-changing gap function.

Next, we test an Einstein local phonon (all-momentum-scattering phonon), i.e., $$\sqrt{{N}_{e}{N}_{h}}{V}_{ph}^{he(eh)}\,=$$$${N}_{e(h)}{V}_{ph}^{ee(hh)}=-\,0.5$$. The calculated *T*_*c*_ is plotted in Fig. 2(b) as the red diamond symbols. Surprisingly it shows that the local phonon also always enhances *T*_*c*_ from $${T}_{c}^{0}$$ for all values of *ε*_*b*_. To see more details, the net phonon boost effect $${\rm{\Delta }}{T}_{c}={T}_{c}-{T}_{c}^{0}$$ (blue squares) is plotted. It is about the same magnitude as the pure forward-scattering phonon case except the region where *ε*_*b*_ is small. When *ε*_*b*_ is small, the sizes of the OPs are close each other as $$|{{\rm{\Delta }}}_{h}^{+}| \sim |{{\rm{\Delta }}}_{e}^{-}|$$, so that the local phonon contribution in Eq. (8) becomes close to 0, hence the *T*_*c*_-boost effect of a local phonon is very weak. As *ε*_*b*_ increases, the net phonon boost effect $${\rm{\Delta }}{T}_{c}={T}_{c}-{T}_{c}^{0}$$ increases until it reaches the maximum and eventually decreases. Δ*T*_*c*_ increases because the gap size disparity $$|{{\rm{\Delta }}}_{h}^{+}|/|{{\rm{\Delta }}}_{e}^{-}|$$ increases as the hole band sinks deeper. Beyond crossing a certain depth as $${\varepsilon }_{b} > {\varepsilon }_{b}^{\ast }$$ (the value $${\varepsilon }_{b}^{\ast }$$ where $${T}_{c}^{0}$$ approaches zero), both Δ*T*_*c*_ and *T*_*c*_ itself decreases because the absolute phase space for the pairing interaction shrinks to zero except the intra-electron-band scattering. This is a totally unexpected result contrary to the common belief that *“the forward-scattering is the necessary condition for the phonon boost effect.”* Our result of Fig. 2(b) is a clear demonstration that any type of phonon should contribute to enhance *T*_*c*_, and the sunken band in the incipient band superconductor plays an active role for this unusual behavior.

**Figure 3:**
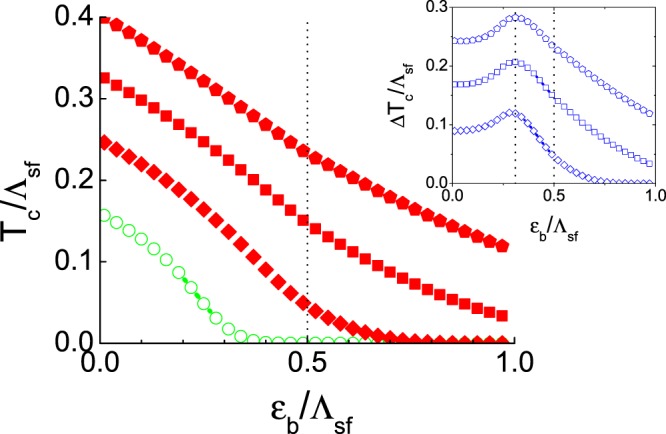
The forward-scattering phonon boost effect. The repulsive spin-fluctuation mediated interaction $\sqrt{{N}_{e}{N}_{h}}{V}_{sf}^{he(eh)}=1.5$ and ${N}_{e(h)}{V}_{sf}^{ee(hh)}=0.4$ is fixed for all calculations. The forward-scattering phonon interaction is varied as ${N}_{e}{V}_{ph}^{ee(hh)}=0.0,-\,0.5,-\,1.0$, and −1.5, respectively, in increasing order of *T*_*c*_, and the inter-band phonon interaction $\sqrt{{N}_{h}{N}_{e}}{V}_{ph}^{he(eh)}=0.0$ for all cases. The inset is the plot of the net phonon boost effect ${\rm{\Delta }}{T}_{c}={T}_{c}-{T}_{c}^{0}$. Vertical lines of ${\varepsilon }_{b}^{\ast }=0.32{{\rm{\Lambda }}}_{sf}$ and *ε*_*b*_ = *ω*_*D*_ = 0.5Λ_*sf*_ are guides for eyes.

To see more details, we calculated the *T*_*c*_ with the different values of the phonon interaction strength for each case. The strength of the spin-fluctuation interaction ${V}_{sf}^{ab}$ is fixed as the same values used in Fig. 2 in all calculations. Figure 3 is the results of *T*_*c*_ with the forward-scattering phonon. The main panel is the calculated *T*_*c*_ with different values of the forward-scattering phonon interaction as ${N}_{e}{V}_{ph}^{ee(hh)}=0.0,-\,0.5,-\,1.0$, and −1.5, respectively, in increasing order of *T*_*c*_, and $\sqrt{{N}_{h}{N}_{e}}{V}_{ph}^{he(eh)}=0.0$ for all cases. The inset of Fig. 3 is the net phonon-boost effect ${\rm{\Delta }}{T}_{c}={T}_{c}-{T}_{c}^{0}$ for each case. The overall behavior is similar in all cases. The magnitude of Δ*T*_*c*_ monotonically increases with the strength of the phonon interaction, and its peak position is always near ${\varepsilon }_{b}^{\ast }$ (see the inset of Fig. 3). On the other hand, there is no noticeable sign in Δ*T*_*c*_ when *ε*_*b*_ crosses the phonon interaction cutoff *ω*_*D*_ = 0.5Λ_*sf*_; this is not the case with the local (all-momentum-scattering) phonon.

**Figure 4:**
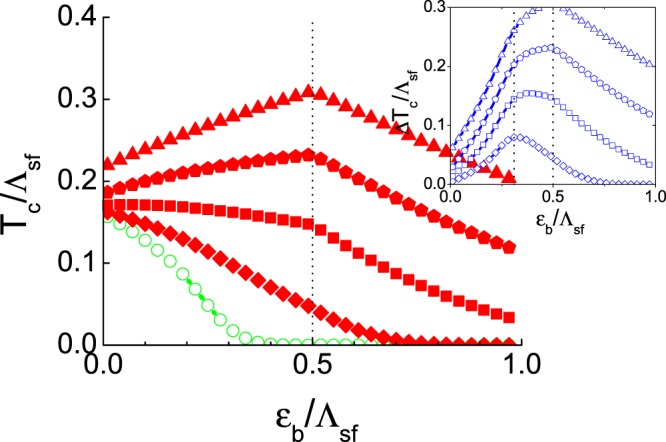
The all-momentum-scattering phonon boost effect. The repulsive spin-fluctuation mediated interaction $\sqrt{{N}_{e}{N}_{h}}{V}_{sf}^{he(eh)}=1.5$ and ${N}_{e(h)}{V}_{sf}^{ee(hh)}=0.4$ is fixed for all calculations. The all-momentum-scattering phonon interaction, ${N}_{e}{V}_{ph}^{ee(hh)}=\sqrt{{N}_{h}{N}_{e}}{V}_{ph}^{he(eh)}={\lambda }_{ph}$, is varied as *λ*_*ph*_ = 0.0, −0.5, −1.0, −1.5, and −2.0, respectively, in increasing order of *T*_*c*_. The inset is the plot of the net phonon boost effect ${\rm{\Delta }}{T}_{c}={T}_{c}-{T}_{c}^{0}$. Vertical lines of ${\varepsilon }_{b}^{\ast }=0.32{{\rm{\Lambda }}}_{sf}$ and *ε*_*b*_ = *ω*_*D*_ = 0.5Λ_*sf*_ are guides for eyes.

Figure 4 is the results of *T*_*c*_ with the all-momentum-scattering phonon, hence ${N}_{e(h)}{V}_{ph}^{ee(hh)}=\sqrt{{N}_{h}{N}_{e}}{V}_{ph}^{he(eh)}={\lambda }_{ph}$ for all calculations. The coupling strength increases as *λ*_*ph*_ = 0.0, −0.5, −1.0, −1.5 and −2.0, respectively, in increasing order of *T*_*c*_. The increased *T*_*c*_ is the similar magnitude as in the case of the froward-scattering phonon except for the small *ε*_*b*_ region. A new finding is that this small *ε*_*b*_ region is defined as ${\varepsilon }_{b} < {\varepsilon }_{b}^{\ast }$ for weak coupling phonon (see the *λ*_*ph*_ = −0.5 data in the inset of Fig. 4). Increasing the coupling strength, this small *ε*_*b*_ region, where Δ*T*_*c*_ is increasing as *ε*_*b*_ increases, extends to *ε*_*b*_ < *ω*_*D*_. This behavior can be clearly seen by comparing the insets of Figs 3 and 4. The Δ*T*_*c*_ of the forward-scattering phonon case in Fig. 3 has always the maximum peak at ${\varepsilon }_{b}={\varepsilon }_{b}^{\ast }\approx 0.32$. On the other hand, the peak position of Δ*T*_*c*_ of the all-momentum-scattering phonon case in Fig. 4 shifts from ${\varepsilon }_{b}={\varepsilon }_{b}^{\ast }\approx 0.32$ to *ε*_*b*_ = *ω*_*D*_ = 0.5 as the phonon coupling increases. As a result, when the phonon coupling strength is strong enough such as *λ*_*ph*_ = −1.5 and −2.0, the increasing slope of Δ*T*_*c*_ is so steep that the total *T*_*c*_ itself develops a maximum peak at *ε*_*b*_ = *ω*_*D*_ and decreases afterwards. This behavior of *T*_*c*_*vs ε*_*b*_ is very different from a standard incipient band model without phonon where *T*_*c*_ monotonically decreases as *ε*_*b*_ increases. Lastly, the *T*_*c*_ boost effect Δ*T*_*c*_ becomes qualitatively the same as in Fig. 3. when *ε*_*b*_ > *ω*_*D*_ (i.e. *ε*_*b*_/Λ_*sf*_ > 0.5). This behavior is natural to understand because the all-momentum-scattering local phonon cannot mediate the inter-band scattering when *ε*_*b*_ > *ω*_*D*_ (see Fig. 1(a)), hence it effectively acts as an “forward-scattering” phonon.

One last remark is that while the calculated *T*_*c*_ in this paper is always of the ${s}_{he}^{+-}$-wave solution as depicted in Fig. 1(b), we have also checked the possibility of the ${s}_{he}^{++}$-wave solution and we found that it never be a solution with all parameter choices of this paper.

## Discussion

We have studied the phonon boost effect on the ${s}_{he}^{+-}$-pairing state of the incipient two band model. We have considered both the forward-scattering phonon and the all-momentum-scattering local phonon. It is confirmed that the forward-scattering phonon is efficient to boost *T*_*c*_. Our model calculations also demonstrated that the optimal condition of the forward-scattering phonon for increasing *T*_*c*_ is to limit its *“forwardness”* narrower than the inter-band distance but wide enough to cover the intra-band scattering of the FSs of each of the electron and hole band.

The most important result of our study is that the all-momentum-scattering local phonon can be an effective *T*_*c*_-booster as much as the forward-scattering phonon. This surprising result is, in fact, a natural consequence of the intrinsic property of the incipient band model, where a normal band (crossing the Fermi level) and an incipient band (sunken below the Fermi level) severely break the balance between the gap sizes of ${{\rm{\Delta }}}_{h}^{+}$ and ${{\rm{\Delta }}}_{e}^{-}$ on each band. This severe gap size disparity turns the all-momentum-scattering local phonon into an effective forward-scattering phonon. Since this gap size disparity is growing as the incipient band sinks deeper, the phonon-boost effect of the local phonon increases as *ε*_*b*_ increases until *ε*_*b*_ reaches to ${\varepsilon }_{b}^{\ast }$ or *ω*_*D*_ depending on the phonon coupling strength.

Finally, our finding sheds a completely new light on the role of phonon interaction in the Fe-based superconductors, in particular, in the heavily electron-doped iron selenide (HEDIS) compounds. The immediate implication to the FeSe/STO monolayer system is that all interface phonons^12^ (both 90 *meV* and 60 *meV* F-K phonons) from the STO substrate–regardless of being forward-scattering phonon or not–as well as all intrinsic phonons inside the FeSe-layer itself should contribute to increasing *T*_*c*_ of the FeSe/STO monolayer system, if the pairing gap symmetry is the ${s}_{he}^{+-}$-wave state. Besides the FeSe/STO monolayer system, other HEDIS compounds such as A_*x*_Fe_2−*y*_Se_2_ (A = K, Rb, Cs, Tl, etc.) (*T*_*c*_ ≈ 30–40 *K*)^16–18^ and (Li_1−*x*_Fe_*x*_OH)FeSe (*T*_*c*_ ≈ 40 *K*)^19^, which all develop a deeply sunken (*ε*_*b*_ ~ 60–90 *meV*) incipient band by electron doping, should also have the phonon-boost effect from the intrinsic phonons in the bulk regardless of their “forwardness” or “local” characters. Our theory can be tested by the isotope effect measurement of the major phonon(s) with O isotope on SrTiO_3_ substrate and Se isotope for FeSe bulk. Lastly, but not least, although the current paper has studied the electron doped system, i.e., HEDIS, the phonon boost mechanism found in this paper should equally apply to the heavily hole doped system, too, because the principle of the all-phonon boost effect on the ${s}_{he}^{+-}$-pairing state relies only on the disparity between the hole band and the electron band.
